# A multivariate analysis of serum nutrient levels and lung function

**DOI:** 10.1186/1465-9921-9-67

**Published:** 2008-09-29

**Authors:** Tricia M McKeever, Sarah A Lewis, Henriette A Smit, Peter Burney, Patricia A Cassano, John Britton

**Affiliations:** 1 Division of Epidemiology and Public Health, University of Nottingham, Nottingham, UK; 2 Centre of Prevention and Health Services Research, National Institute of Public Health, Bilthoven, The Netherlands; 3 Peter Burney, Respiratory Epidemiology & Public Health, Imperial College, London, UK; 4 Division of Nutritional Sciences, Cornell University, Ithaca, USA; 5 Division of Epidemiology and Public Health, Clinical Science Building, City Hospital, Hucknall Road, Nottingham, NG5 1PB, UK

## Abstract

**Background:**

There is mounting evidence that estimates of intakes of a range of dietary nutrients are related to both lung function level and rate of decline, but far less evidence on the relation between lung function and objective measures of serum levels of individual nutrients. The aim of this study was to conduct a comprehensive examination of the independent associations of a wide range of serum markers of nutritional status with lung function, measured as the one-second forced expiratory volume (FEV_1_).

**Methods:**

Using data from the Third National Health and Nutrition Examination Survey, a US population-based cross-sectional study, we investigated the relation between 21 serum markers of potentially relevant nutrients and FEV_1_, with adjustment for potential confounding factors. Systematic approaches were used to guide the analysis.

**Results:**

In a mutually adjusted model, higher serum levels of antioxidant vitamins (vitamin A, beta-cryptoxanthin, vitamin C, vitamin E), selenium, normalized calcium, chloride, and iron were independently associated with higher levels of FEV_1_. Higher concentrations of potassium and sodium were associated with lower FEV_1_.

**Conclusion:**

Maintaining higher serum concentrations of dietary antioxidant vitamins and selenium is potentially beneficial to lung health. In addition other novel associations found in this study merit further investigation.

## Background

Chronic obstructive pulmonary disease (COPD) is a common disease characterised by reduced FEV_1_. Although smoking is the main identified risk factor for COPD it is clear that other aetiological factors are also involved. There is now substantial observational evidence, based predominantly on food frequency questionnaire measures of intake, that a diet high in antioxidants is associated with better lung function [1-4]. However, a major randomized controlled trial of supplementation with the main antioxidant vitamins C, E, and beta-carotene recently failed to identify any beneficial effect on COPD outcomes [5]. One possibility is that the effects of these particular nutrients operate at an earlier point in the natural history of COPD, or that the observational evidence is confounded by the effects of other nutrients or lifestyle factors, or it is possible that these nutrients do not have universal benefit and only certain subgroups would benefit from supplementation.

Much of the available epidemiological evidence is based on findings using food frequency questionnaires to assess diet. This method of assessing nutritional status has potential limitations[6]. Serum nutrient levels provide an alternative and objective measure of nutritional status, but there are relatively few studies of the relation between nutrients and lung function available [7-15] and these have generally involved relatively small numbers of subjects or else have studied the effects of only a limited number of nutrients.

The aim of this study was therefore to use the comprehensive data from the Third National Health and Nutrition Survey (NHANES III) to extend an earlier investigation of 4 antioxidants (vitamin C, vitamin E, β-carotene, and selenium) and lung function[7], and in addition, to investigate the association of novel serum markers in relation to lung function, measured as one-second forced expiratory volume (FEV_1_), in an exploratory analyses.

## Materials and methods

Between 1988 and 1994, a survey was conducted to examine the health and nutrition of a randomly selected sample of the non-institutionalized US population. Full details of the survey design and examination procedure have been previously published[16]. This study examines adults aged 17 and older, which yields a study sample population of 20,050. However, exclusions from the study sample including missing data on lung function, missing data on most of the exposure variables, or on any confounding variables in the final model, resulted in a final sample size of 14,120.

### Data collection

Trained interviewers collected detailed information on socioeconomic and medical history questionnaires on each participant, including questions on social class, smoking history, medical diagnosis, and current medication. Further measurements were conducted at mobile examination centers, including anthropometric measurements, which were used to calculate body mass index (BMI (weight (kg) divided by height (m) squared)) and waist to hip ratio (WHR). Complete medical examinations were conducted and blood samples were collected for a variety of biochemical assays, including vitamins (vitamin A, alpha-carotene, beta-carotene, beta-cryptoxanthin, lutein/zeaxanthin, lycopene, retinyl esters, vitamin B12, red blood cell folate, vitamin C, and vitamin E), minerals (selenium, normalised calcium, chloride, iron, total iron binding capacity(TIBC), ferritin, transferrin saturation, potassium, and sodium), total cholesterol, triglycerides and total protein[17]. As part of the medical examination, spirometry measurements including FEV_1 _and forced vital capacity (FVC) were conducted according to the guidelines of the American Thoracic Society and the highest value from the acceptable manoeuvres was recorded. The present study has used the one-second forced expiratory volume (FEV_1_) as its primary lung function outcome variable.

### Statistical analyses

Self-reported smoking history was used to categorize participants into never smokers, ex-smokers, and current smokers. Data on cigarette consumption were used to determine pack-years and prolonged periods in which a person had quit smoking were accounted for in determining pack-years. BMI was also categorised into underweight (BMI < 20), normal (≥ 20 BMI < 25), overweight (≥ 25 BMI < 30) and obese (BMI ≥ 30). A variety of models for FEV_1 _were examined including ones with interaction and higher order terms and as the results were similar for all of them and the model fit was only marginally better with the additional terms the simplest baseline model was chosen which included age, sex, height, smoking (status and pack-years), and race/ethnicity. In models including fat soluble vitamins, serum triglycerides and total cholesterol were additionally included in the model to adjust for their confounding effect. Serum nutrient values were divided into quintiles and fitted as ordered categorical variables and unordered dummy variables to assess the linearity of the relation. Nutrients showing a linear association with FEV_1 _were then included in the analysis as continuous variables and their effects calculated as change in FEV_1 _(in mL) per standard deviation (SD) change in nutrient level. Those showing nonlinear effects were modelled as categorical quintile variables. We examined the correlation matrix and took this into account in subsequent modelling.

In our analyses we first divided the nutrients into 2 groups; an antioxidant group including vitamins and selenium, all of which are potentially involved in antioxidant defences, and a more diverse group of nutrients and biological mineral levels that have previously been or could potentially be implicated in lung disease but with less clearly established mechanisms of effect. We explored independent effects initially within these two groups, first modelling each nutrient alone to determine its unique association with FEV_1 _(Model 1). Next using backward and forward modelling, a mutually adjusted model was created and then simplified to only include those variables that had statistically significant associations with FEV_1_(Model 2). Finally serum nutrient biomarkers were combined across vitamins and minerals for a fully adjusted model (Model 3). We also retained nutrients in Model 2 if they had an independent, statistically significant association with FEV_1 _in Model 3. We investigate whether these models were affected by the correlation between serum nutrients, and examined final models for their validity of estimates given the potential effects of multi-collinearity.

We investigated a number of potential confounding effects including BMI, WHR, poverty index ratio, level of education, physical activity, energy intake, passive smoking, C-reactive protein and co-morbid conditions (including heart disease, cancers, diabetes, and other conditions). As there was a priori evidence to suggest that there may be differences in associations according to smoking status, we looked for evidence of effect modification by smoking status and sex (in Model 1) on the individual nutrient effects identified in Model 3. In addition, we examined the data allowing for the multiple testing using Bonferroni correction to the p-values. All results presented were conducted whilst accounting for the complex, multi-stage probability sample design of NHANES III and all data were analyzed using STATA SE 9.0 (Stata Corporation, Texas).

## Results

There were 6,671 (47.3%) males in the study population and 7,449 (52.8%) females (Table 1). Analysis of available data for participants excluded as a result of incomplete data indicated that they were slightly older, with a mean age of 52.0 as compared to 45.7, and included a slightly higher proportion of ex-smokers, but appeared otherwise to be broadly similar to those with complete data. Demographic data were similar also for the study population with data available for vitamin B12 (n = 7360) and normalised calcium (n = 12657). Mean serum nutrient levels and their standard deviations are shown in Table 2.

**Table 1:** Demographics and characteristics of the study population and those subjects excluded from the study*

**Variable**	**Participants included** N = 14120		**Participants excluded** N = 5930	
	Mean (SD)	Number (%)	Mean (SD)	Number (%)

**Sex**				
Males		6671 (47.3)		2730 (47.8)
Females		7449 (52.8)		3200 (54.0)
**Age**	45.7 (19.6)		52.0 (22.8)	
**Smoking status**				
Never		7390 (52.3)		2845 (48.1)
Ex		3174 (22.5)		1633 (27.6)
Current		3556 (25.2)		1434 (24.3)
Pack Years**	0 (0 to 12)		0 (0 to 14)	
**Race/Ethnicity**				
Non-Hispanic White		5881 (41.6)		2602 (43.9)
Non-Hispanic Black		3860 (27.3)		1626 (27.4)
Mexican-American		3802 (26.9)		1504 (25.4)
Other		577 (4.1)		198 (3.3)
**FEV**_1 _(L)	3.0 (0.9)		2.8 (1.0)	
**FVC **(L)	3.8 (1.1)		3.6 (1.1)	
**BMI **(kg/m^2^)	27.0 (5.8)		26.6 (6.2)	
**Cholesterol **(mmol/l)	5.3 (1.2)		5.3 (1.2)	
**Triglycerides **(mmol/l)	1.6 (1.3)		1.7 (1.3)	
**Energy intake **(kcal)	2102 (1068)		2006 (1039)	
**C-reactive protein **(mg/dL)**	0.21 (0.21 to 0.40)		0.21 (0.21 to 0.51)	
**Activity level**				
None		2800 (19.8)		1886 (31.8)
Low		5867 (41.6)		2012 (33.9)
Moderate		4579 (32.4)		1741 (29.4)
High		874 (6.2)		291 (4.9)

* Data is presented for the population where data is available. Excluded participants had missing data on a priori confounders and/or serum markers

** Data presented as Median and IQR

**Table 2:** Mean levels of serum nutrients in the study population

**Nutrient**	**Mean**	**SD**
**Antioxidants**		
Vitamin A (μmol/L)	1.98	0.58
Alpha-carotene (μmol/L)	0.08	0.10
Beta-carotene (μmol/L)	0.37	0.40
Beta-cryptoxanthin (μmol/L)	0.19	0.15
Lutein/zeaxanthin (μmol/L)	0.40	0.23
Lycopene (μmol/L)	0.41	0.21
Retinyl Esters (μmol/L)*	0.19	0.15
Vitamin B12 (pmol/L)	444.9	1913.8
Vitamin C (mmol/L)	40.2	25.4
Vitamin E (μmol/L)	26.0	11.6
Selenium (nmol/L)	1.6	0.2

**Minerals and other nutrients**		

Normalised calcium (mmol/L)**	1.24	0.05
Chloride (mmol/L)	104.5	3.3
Iron (μmol/L)	15.7	6.7
TIBC (μmol/L)	63.5	10.4
Transferrin saturation (%)	25.4	11.4
Ferritin (μg/L)	129.3	143.8
Red blood cell folate (nmol/L)	420.1	229.5
Potassium (mmo/L)	4.1	0.3
Sodium (mmol/L)	141.3	2.4

Total Protein (g/L)	74.0	5.0

* Data available only for 7360 participants

**Data available only for 12657 participants

In models considering single nutrients in the antioxidant group, vitamin A, retinyl esters, alpha-carotene, beta-carotene, beta-cryptoxanthin, lutein/zeaxanthin, lycopene, vitamin C, vitamin E and selenium were each associated with FEV_1 _(Table 3, Model 1). In the mutually adjusted Model 2, some of these regression coefficients were attenuated; however vitamin A retained a relatively strong association with FEV_1 _(increase per standard deviation increase in vitamin A = 31.2 mL, 95% CI 21.8 to 40.5), as did selenium, the difference in FEV_1 _between persons in the highest vs. lowest quintiles was 60.1 mL (95% CI 34.0 to 86.2). There was little or no change in these regression coefficients after further adjusting for serum markers that had statistically significant effects in the minerals and other nutrients regression model (Table 3, Model 3).

**Table 3:** Difference in FEV1 for a one SD or quintile increase in antioxidants

**Nutrient**	**Model as**	Model 1*		**Model 2†**		**Model 3‡**	
		
		**β coeff**	**95% CI**	**β coeff**	**95% CI**	**β coeff**	**95% CI**
Vitamin A (μmol/L)	Per SD change	42.6	32.4 to 52.9	31.2	21.8 to 40.5	33.1	23.7 to 42.6
							p < 0.001

Alpha-carotene (μmol/L)	Per SD change	23.7	6.4 to 41.1				

Beta-carotene (μmol/L)	Per SD change	25.5	16.3 to 34.7				

Beta-cryptoxanthin (μmol/L)	≤ 0.09	reference		reference		reference	
	0.10 – 0.13	44.9	19.4 to 70.4	20.9	-3.4 to 45.3	26.4	-0.3 to 53.0
	0.14 – 0.18	93.6	68.0 to 119.3	57.1	31.2 to 82.9	57.1	30.8 to 83.5
	0.19 – 0.25	94.6	63.4 to 125.9	50.7	22.6 to 78.8	60.4	25.7 to 95.0
	≥ 0.26	110.7	78.2 to 143.2	52.3	22.1 to 82.5	66.3	31.5 to 101.6
							p = 0.004

Lutein/Zeaxanthin (μmol/L)	Per SD change	29.2	16.2 to 42.3	14.1	4.6 to 23.6	8.6	-1.5 to 18.6
							p = 0.092

Lycopene (μmol/L))	≤ 0.22	reference		reference		reference	
	0.23 – 0.34	52.8	25.1 to 80.6	35.9	11.3 to 60.5	33.8	5.2 to 62.5
	0.35 – 0.43	63.7	39.8 to 87.6	39.2	12.1 to 66.2	35.6	10.0 to 61.1
	0.44 – 0.58	70.5	40.1 to 100.9	40.0	13.4 to 66.5	36.9	7.9 to 65.9
	≥ 0.59	88.0	59.9 to 116.2	48.9	19.9 to 77.9	54.3	25.0 to 83.6
							p = 0.01

Retinyl Esters (μmol/L)	Per SD change	23.5	11.9 to 35.0				

Vitamin B12 (pmol/L)	≤ 239.1	reference					
	239.2 – 304.0	4.4	-30.2 to 39.1				
	304.1 – 374.8	-9.3	-43.7 to 25.0				
	374.9 – 478.1	-7.2	-42.0 to 27.5				
	≥ 478.2	-29.7	-64.5 to 5.2				

Vitamin C (mmol/L)	Per SD change	38.1	28.1 to 48.0	17.0	6.8 to 27.3	17.9	7.5 to 28.2
							p < 0.001

Vitamin E (μmol/L)	Per SD change	45.6	32.9 to 58.3	23.1	11.5 to 34.7	25.3	12.3 to 38.4
							p < 0.001

Selenium (nmol/L)	≤ 1.4	reference		reference		reference	
	1.41 – 1.5	50.0	23.5 to 76.6	40.5	15.5 to 65.5	43.4	13.6 to 73.1
	1.51 – 1.6	61.1	33.8 to 88.4	48.5	23.8 to 73.3	57.0	29.5 to 84.5
	1.61 – 1.73	89.9	61.1 to 118.7	73.1	48.0 to 98.3	76.7	47.1 to 106.4
	≥ 1.74	80.4	50.5 to 110.2	60.1	34.0 to 86.2	68.6	34.8 to 102.4
							0.002

*Model 1- Adjusted for age, sex, height, smoking (status and packyears), BMI, race/ethnicity and fat-soluble vitamins adjusted for cholesterol and triglycerides, considering the nutrients individually

**†**Model 2- Adjusted for covariates listed under Model 1, as well as for all nutrients with statistically significant associations with lung function in a mutually adjusted model. Number of participants in model = 14120

**‡**Model 3- Adjusted for all covariates and nutrients in Model 1 & 2 and additionally adjusted for minerals and other nutrients found to be significantly associated with lung function (model 2) in Table 4. Number of participants in model = 12657

In univariate analysis of minerals and other nutrients, normalised calcium, chloride, iron, transferrin saturation, red blood cell folate, potassium, sodium and total protein were statistically significantly associated with FEV_1 _(Table 4, Model 1). In the mutually adjusted Model 2, normalised calcium had an inverse U-shaped relation with FEV_1_, as the third and fourth quintiles were associated with better lung function compared to the second and fifth quintiles. A higher concentration of serum chloride was associated with higher FEV_1 _(FEV_1 _difference per standard deviation increase in chloride = 35.6 mL, 95% CI 22.8 to 48.5). Although there was not a clear dose-response relation, serum iron also had a positive association with FEV_1_, such that persons in the highest quintile of iron had an average FEV_1 _that was 77.8 ml higher (95% CI 45.5 to 110.0) than persons in the lowest quintile. There was a very strong correlation between iron and transferrin saturation (r = 0.92) and when put in Model 2 without iron in the model, each standard deviation increase in transferrin saturation was associated with a 20.9 mL (95% 10.9 to 30.9) increase in FEV_1_. Serum potassium had an inverse association with FEV_1_, the FEV_1 _difference per standard deviation change in potassium was -15.6 mL (95% CI -22.1 to -9.0), and there was also an inverse association with sodium (FEV_1 _difference per standard deviation = -10.1 mL, 95% CI -21.0 to 0.72). Associations in the mineral and other nutrient group were not appreciably altered by adjusting for antioxidant nutrients.

**Table 4:** Difference in FEV1 for a one SD or quintile increase in minerals and other nutrients

**Nutrient**	**Model as**	Model 1*		**Model 2†**		**Model 3‡**	
		
		**β coeff**	**95% CI**	**β coeff**	**95% CI**	**β coeff**	**95% CI**
Normalised calcium (mmol/l)	≤ 2.23	reference		reference		reference	
	2.24 – 2.29	38.9	11.9 to 65.9	41.2	14.8 to 67.5	36.5	10.3 to 62.7
	2.30 – 2.33	68.5	43.9 to 93.0	71.8	47.1 to 96.5	64.1	39.5 to 88.6
	2.34 – 2.39	50.3	19.6 to 80.9	58.5	28.3 to 88.6	50.3	19.6 to 80.9
	≥ 2.4	25.8	-6.5 to 58.0	39.4	7.1 to 72.5	29.0	-1.0 to 52.7
							p = 0.001

Chloride (mmol/L)	Per SD change	27.2	17.9 to 36.5	35.6	22.8 to 48.5	40.5	28.4 to 52.7
							p < 0.001

Iron (μmol/L)	≤ 10.21	reference		reference		reference	
	10.22 – 13.43	33.6	8.9 to 58.3	37.5	11.5 to 63.5	23.3	-3.6 to 50.2
	13.44 – 16.48	68.0	43.1 to 92.8	72.2	46.1 to 98.2	51.0	23.9 to 78.2
	16.49 – 20.6	51.8	29.1 to 74.6	58.0	35.5 to 80.5	35.2	12.9 to 57.6
	≥ 20.61	70.8	40.1 to 101.6	77.8	45.5 to 110.0	54.2	21.0 to 86.4
							p = 0.0054

TIBC (μmol/L)	≤ 54.98	reference					
	54.99 – 60.36	12.8	-13.0 to 38.6				
	60.37 – 65.19	19.1	-2.9 to 41.0				
	65.20 – 71.82	3.1	-24.9 to 31.0				
	≥ 71.83	-25.4	-52.7 to 1.9				

Transferrin saturation (%)	Per SD change	24.2	14.2 to 34.2				

Ferritin (μmol/L)	Per SD change	3.4	-5.1 to 12.0				

Red blood cell folate (nmol/L)	≤ 256.1 256.2 – 326.3	reference 40.4	16.9 to 63.8	reference 41.1	16.8 to 65.3	reference 27.4	3.9 to 51.0
	326.4 – 407.9	33.3	4.5 to 62.1	29.7	1.0 to 58.4	9.3	-18.2 to 36.8
	408.0 – 555.2	43.3	16.9 to 69.7	45.5	17.1 to 73.9	14.1	-13.9 to 42.2
	≥ 555.3	36.3	8.0 to 64.6	34.3	2.8 to 65.8	-13.2	-45.1 to 18.7
							p = 0.0207

Potassium (mmol/l)	Per SD change	-10.7	-18.2 to -3.1	-15.6	-22.1 to -9.0	-21.2	-28.3 to -14.1
							p < 0.001

Sodium (mmol/l)	Per SD change	6.6	-0.9 to 14.0	-10.1	-21.0 to 0.72	-13.0	-24.1 to -2.0
							p = 0.022

Total Protein (g/L)	Per SD change	-17.2	-28.5 to -5.9				

* Model 1- Adjusted for age, sex, height, smoking (status and packyears), BMI, race/ethnicity

**† **Model 2- Adjusted for covariates listed under Model 1, as well as for all nutrients with statistically significant associations with lung function in a mutually adjusted model. Number of participants in model = 14120

**‡ **Model 3- Adjusted for all covariates and nutrients in Model 1 & 2 and additionally adjusted for minerals and other nutrients found to be significantly associated with lung function (model 2) in Table 3. Number of participants in model = 12657

We investigated potential confounding by a number of other factors including waist to hip ratio (WHR), poverty index ratio, level of education, physical activity, energy intake, passive smoking, C-reactive protein and co-morbid illness. The further consideration of these variables had no notable effect on the estimates: the majority of model coefficients were within 5% of their original value when further variables were added to the model. We also looked for evidence of effect modification by smoking status and found statistical evidence for a smoking by nutrient interactions for vitamin A, lycopene, red blood cell folate, chloride and vitamin E. Lycopene and red blood cell folate did not have a consistent association pattern across smoking categories, whereas vitamin A, chloride, and vitamin E all showed a stronger association with FEV_1 _among current smokers (Table 5). We have examined the data for interactions with sex, and found significant interactions for lycopene, selenium and chloride, all of which had a greater effect in men than in women (data not shown).

**Table 5:** Stratified analyses of smoking with certain nutrients*

**Nutrient**		**Non-smokers**		**Ex-Smokers**		**Current Smokers**	
		**β coeff**	**95% CI**	**β coeff**	**95% CI**	**β coeff**	**95% CI**

Vitamin A (μmol/L)	Per SD change	15.9	-0.8 to 32.6	24.6	6.3 to 42.9	51.8	35.4 to 68.3
							p < 0.001

Lycopene (μmol/L)	≤ 0.22	reference		reference		reference	
	0.23 – 0.34	55.8	26.1 to 85.5	0.4	-60.8 to 61.6	26.3	-29.0 to 81.6
	0.35 – 0.43	45.4	12.9 to 77.9	31.0	-29.7 to 91.7	15.2	-44.4 to 74.8
	0.44 – 0.58	37.7	-1.7 to 77.2	25.2	-24.3 to 74.6	50.1	-9.4 to 109.6
	≥ 0.59	52.3	13.0 to 91.7	92.0	31.3 to 152.7	31.4	-28.3 to 91.1
							p = 0.59

Vitamin E (μmol/L)	Per SD change	17.8	1.8 to 33.9	28.4	12.1 to 44.8	39.7	9.5 to 69.9
							p = 0.011

Chloride (mmol/L)	Per SD change	29.8	16.2 to 43.3	51.0	28.0 to 73.9	59.4	29.8 to 89.0
							p < 0.001

Red blood cell folate (nmol/L)	≤ 256.1	reference		reference		reference	
	256.2 – 326.3	28.5	-8.9 to 61.8	23.7	-49.0 to 96.5	34.4	-24.5 to 93.3
	326.4 – 407.9	5.0	-34.7 to 44.8	36.7	-28.7 to 102.8	-0.7	-48.0 to 46.6
	408.0 – 555.2	12.7	-25.9 to 51.3	3.3	-69.3 to 75.9	28.8	-30.3 to 88.0
	≥ 555.3	-0.3	-42.7 to 42.6	-22.9	-99.2 to 53.3	-0.8	-76.2 to 74.6
							p = 0.58

* adjusted for age, sex, height, packyears (where appropriate), BMI, race/ethnicity and fat-soluble vitamins adjusted for cholesterol, triglycerides, and other important nutrients

Finally, we conducted sensitivity analyses to examine whether the results were similar after the exclusions of selected participants. When the results were examined excluding individuals who used vitamin or mineral supplements (n = 5149, 36%), the majority of the results were similar; increases in the effect size were seen for vitamin E and iron, whereas the effect sizes for lycopene and selenium were slightly reduced. Excluding people with asthma (n = 991, 7%) from the study population did not alter the effect estimates. Excluding participants with COPD (n = 989, 9%) [self-reported physician-diagnosed emphysema and/or chronic bronchitis, and/or by GOLD spirometry criteria (FEV_1_/FVC < 70% and FEV_1 _< 80% predicted although not post-bronchodilator)], yielded effect sizes that were reduced slightly, but did not affect the overall conclusions of the analysis. Similar conclusions were made when lung function was modelled as FVC.

When we examined the correlation matrix between serum nutrients the vast majority had very weak correlations, and the only strong correlation (r > 0.6) was found between iron and transferrin saturation: these two nutrients were never included in the same model. Within Model 3, 95% of the correlations between nutrients were less than 0.3 and the strongest correlation found was between lutein/zeaxanthin and beta-cryptoxanthin (r = 0.50), however modelling them separately did not alter findings; it only increased the size of the effects in the final model. The final model was examined for collinearity and there was no strong evidence of collinearity within the model as all of the variance inflation factors were less than 5 and the mean variation inflation factor was 1.86. Lastly, if we apply the Bonferoni correction to the p-values, then only p-values of less than 0.002 would be considered as statistically significant. This multiple comparison approach would have excluded the following nutrients from the final model lutein/zeaxanthin (p = 0.092), lycopene (p = 0.01) iron (p = 0.005), red blood cell folate (p = 0.021), and sodium (p = 0.022).

## Discussion

There is already an extensive literature on the relation between measures of dietary intake, lung function and various other respiratory disease outcomes, which has been reviewed elsewhere[1,2,4,18,19], but relatively few of the available studies have explored the effects of serum nutrient markers. Most previous studies have also investigated the effects of a particular nutrient or nutrient group, and are thus open to error arising from confounding effects of other nutrients. In this study we have used the extensive NHANES III dataset in a systematic analysis of all available levels of nutrients and minerals available within the dataset and have used a stepwise grouped analysis to identify independently significant effects on FEV_1_. The *a priori *objective of this study was to test all available serum nutrients in the NHANES III dataset in a single model, to identify the relative importance of individual nutrients, and the statistical power available in the dataset has allowed us to distinguish independent effects of several exposures. In addition, the majority of the nutrients had very weak correlations with other serum nutrients. A systematic approach to modelling was taken and one of the explicit goals of the analyses was to discover new associations. Most of the p-values were very small (p < 0.002), however if we applied the conservative approach of the Bonferroni correction, 4 of the nutrients in the final model would no longer be considered statistically significant. However, the results from this approach must also be interpreted with caution due to the potential of type II error[20]. Similar to previous studies, the effects of some of antioxidants were stronger in smokers as compared to non-smokers. Although the levels of serum markers in males and females were similar, the effect of a few of the antioxidants appeared to be stronger in males compared to females, and these interactions need to be confirmed in other datasets.

Although all of the nutrient levels we analysed are dependent at least to some degree on dietary intake, some (such as sodium and calcium) are closely regulated by homeostatic systems in the body, so in these and in some other cases levels are likely to be low only when intake is extremely low. However we have included these nutrients in the analysis since all have potential links with lung defences, airway calibre or other factors relevant to COPD. In addition, for the majority of study participants, the diet will tend to track through their lifetime, and therefore these cross-sectional relations are important to investigate.

We found that in our mutually adjusted models, FEV_1 _was independently and directly related to levels of vitamin A, beta-cryptoxanthin, lutein/zeaxanthin, lycopene, vitamin C, vitamin E, selenium, normalised calcium, chloride and iron, and was inversely related to potassium and sodium. Of nutrients with linear associations with FEV_1 _the strongest effects per standard deviation change were evident for chloride and vitamin A. Of variables with non-linear associations, the strongest category effects were seen with beta-cryptoxanthin and selenium.

Our findings for the nutrients in the antioxidant group were predictably similar to previous findings from a less extensive analysis of data from NHANES III [7], with vitamin C, vitamin E and selenium identified as independent predictors of FEV_1_, but after adjustment for these nutrients the effect serum beta-carotene was not independently associated with lung function in both analyses of the data. If lung function is modelled in a similar fashion to the prior paper, the effect sizes for vitamin C, vitamin E and selenium are similar to the previously published mutually-adjusted model even after adjustment for the other serum antioxidants that were associated with lung function. This finding is consistent with a relatively predominant role of vitamin C in serum and interstitial fluid in maintaining membrane-bound vitamin E in a reduced state [21]. Three other studies of either serum or plasma vitamin C have reported a protective effect on FEV_1_[9,13,15], though this was not confirmed in one study [8]. There is less evidence of a positive relation between serum vitamin E and FEV_1 _[8], with the majority studies finding no association [9,10,15,22]. Previous results have found protective effects for vitamin A, beta-cryptoxanthin, retinol, total carotenoids, alpha-carotene and beta carotene [8,10,12,14,22].

A growing body of evidence suggests that higher levels of selenium are associated with a reduced risk of asthma [23-30], but the evidence on the relation of selenium to COPD is much more limited. One other population-based study in Nottingham, UK has investigated this association and found that higher levels of serum selenium were associated with higher lung function [15]. The role of selenium in antioxidant defence through the glutathione peroxidase activity is now well established however, and it is therefore plausible that selenium intake is an important determinant of lung defence against damage from cigarette smoke and other environmental pollutants contributing to the aetiology of COPD. The role of selenium therefore deserves further study in randomised controlled trials.

Our analysis of mineral effects found strong effects of serum chloride and sodium on FEV_1_, and we are not aware of any previous reports of these associations. There is substantial literature suggesting an association between sodium intake and self-reported asthma and/or other respiratory symptoms [31-33], exercise induced asthma [34-37], and airway hyper responsiveness[38,39], although not all studies support these findings [31,40-44]. The mechanism of this association in asthma is not understood however, and other studies have not found a relation between sodium intake and FEV_1 _[43,45]. There are no previous reports of an association between serum chloride and FEV_1_, and an intervention study in exercise-induced asthma reported findings that contradict ours, in that dietary chloride was associated with a reduction in lung function after an exercise challenge test [36]. Our findings are based on serum levels of sodium and chloride, which are predominantly under hormonal and renal control and relatively insensitive to dietary intake, but the strength of the associations indicate that they deserve further investigation.

Our finding of a negative association between FEV_1 _and serum potassium level is also, to our knowledge, new. It is also consistent with reported associations between increased urinary potassium and increased airway hyperresponsiveness[31,46] and also lower lung function in girls [45], but two other studies have found no association with serum potassium and asthma [47,48] and one has reported evidence of an opposite effects, such that lower levels of serum potassium were associated with a greater risk of asthma[49].

To our knowledge the associations reported herein between lung function and serum levels of iron, calcium and folate have not previously been reported, though all have biologically plausible effects on the lung. In the case of iron, effects may be mediated through peripheral involvement in antioxidant processes [50-55], whilst a protective effect of calcium could be explained by an interaction with the effects of magnesium, which influence intracellular calcium levels and in so doing affects smooth muscle tone [56]. There is evidence that dietary magnesium has a protective effect on lung function but serum magnesium data were not available in NHANES III [57,58]. Folate has been reported elsewhere to be low in cases of asthma relative to controls[59], and our results generally support that increased red blood cell folate is positively associated with the FEV_1_.

## Conclusion

To summarise, our study confirms that antioxidant levels in blood are associated with higher levels of FEV_1 _and hence may mediate reduced susceptibility to COPD. In addition to the antioxidant vitamins, selenium is also potentially important. Although the effects of the antioxidant vitamins have been recognised for some time, the potential beneficial role of selenium deserves further investigation. Likewise, our finding that sodium, chloride and potassium levels are also related to lung function needs to be tested in other datasets, and if confirmed, the relative importance of intake and homeostatic control mechanisms investigated.

## Abbreviations

FEV1: Forced expiratory volume in 1 second; BMI: Body mass index; COPD: Chronic obstructive pulmonary disease; SD: Standard deviation; WHR: Waist to Hip Ratio; TIBC: Total iron binding capacity.

## Competing interests

The authors declare that they have no competing interests.

## Authors' contributions

TM was responsible for the statistical analyses and draft of the manuscript. SL, HS, PB, PC & JB all contributed to the design of the study and draft of the manuscript. All authors read and approved of the final manuscript.
